# Research on the Friction Noise Generation Mechanism and Suppression Method of Submarine Rubber-Based Propeller Bearings—A Review

**DOI:** 10.3390/polym15163352

**Published:** 2023-08-10

**Authors:** Cunguang Cai, Yuqiang Cheng, Changgeng Shuai

**Affiliations:** 1 Institute of Noise & Vibration, Naval University of Engineering, Wuhan 430033, China; 2 State Key Lab of Ship Vibration & Noise, Wuhan 430033, China

**Keywords:** submarine rubber-based propeller bearing, friction noise generation mechanism, friction noise suppression method

## Abstract

This article introduces the main mechanisms of friction noise generated by submarine rubber-based propeller bearings and analyzes their respective scope of application and limitations. Then, the research on suppressing friction noise through the optimization of the structure and improvement of materials of rubber-based propeller bearings is discussed. Finally, the article summarizes a promising research direction aimed at eliminating friction noise in submarine rubber-based propeller bearings. By improving the structure and materials, the friction noise of propeller bearings can be effectively suppressed, thereby improving the deterrence and stealth performance of submarines.

## 1. Introduction

**Figure 1:**
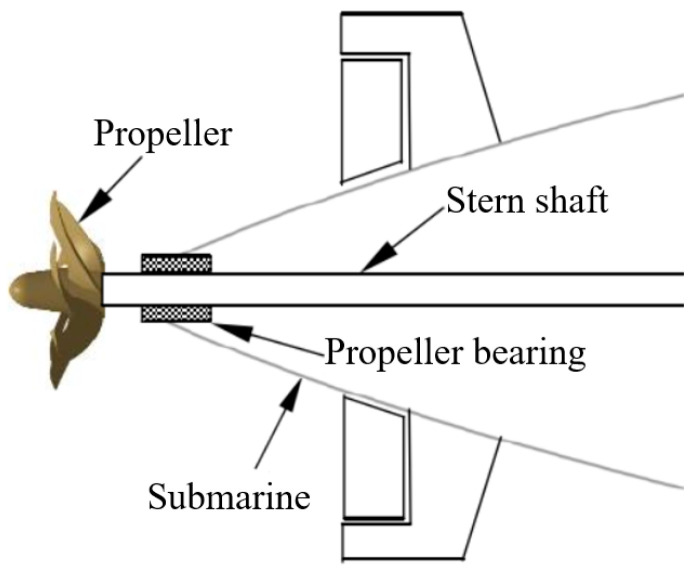
Schematic drawing of submarine propeller bearing.

**Figure 2:**
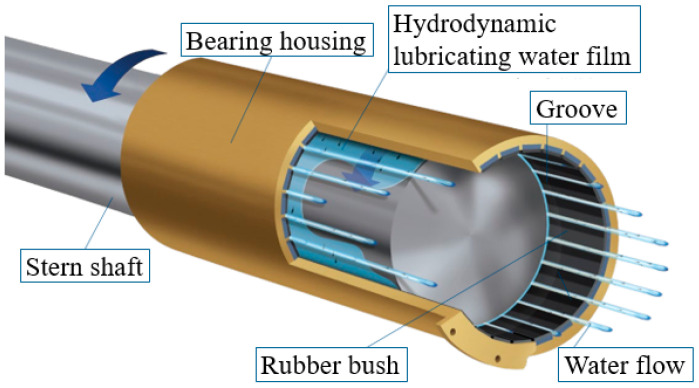
Diagram of rubber-based propeller bearing.

Submarines possess extremely strong stealth and assault capabilities and are recognized as one of the most intimidating and aggressive weapons. All major maritime nations are committed to developing submarines. As shown in Figure 1, the propeller bearing is an important component of the submarine’s shaft system, supporting the propeller shaft and ensuring that the propeller and stern shaft work normally under various operating conditions while isolating the transmission of shaft system vibration to the hull structure. With reference to Figure 2 [1], rubber-based propeller bearings can generate lubricating water film through relative rotation with the shaft in the aquatic environment, greatly reducing wear as well as the friction coefficient (FC) and extending the bearing life. They also have excellent impurity tolerance, outstanding cushioning and vibration-damping performance. They are not sensitive to installation errors, can conform to shaft system bending, have a wide range of material sources, and are low-cost, easy-processing, and environmentally friendly. Therefore, they have been widely used in submarines. In addition, like rubber tires, rubber-based propeller bearings can also be recycled for vibration reduction and ballast protection in railway traffic [2,3,4,5,6,7], which not only reduces economic costs but also contributes to environmental protection.

However, rubber-based propeller bearings still have problems, such as the production of abnormal friction noise during start-up and shut-down, during low-speed and heavy-load operation, or poor lubrication. Once there is abnormal friction noise in the submarine propeller bearing, the submarine’s noise level will suddenly increase, greatly weakening the submarine’s stealth capabilities, reducing its deterrence, and even posing a serious threat to the submarine’s survival. To eliminate the friction noise of submarine rubber-based propeller bearings, many scholars have investigated the mechanism and suppression methods of friction noise.

After decades of research and experimentation following the end of World War II, the U.S. Navy resolved many key technical problems of rubber bearings, such as their material, structure, and lubrication theory. In addition to the United States, countries such as Russia, the United Kingdom, Germany, Japan, Canada, and China have also been conducting in-depth research on noise-reduction technology for submarine propeller bearings. Regarding the friction noise of submarine rubber-based propeller bearings, a large number of research results has been obtained, but due to the complexity of the problem, most of the issues remain in the research stage. This article first discusses the mechanism of friction noise generation in submarine rubber-based propeller bearings, then introduces noise-suppression methods, and finally summarizes and suggests directions for further research on friction noise in submarine rubber-based propeller bearings.

## 2. Friction Noise Generation Mechanism of Submarine Rubber-Based Propeller Bearings

**Figure 3:**
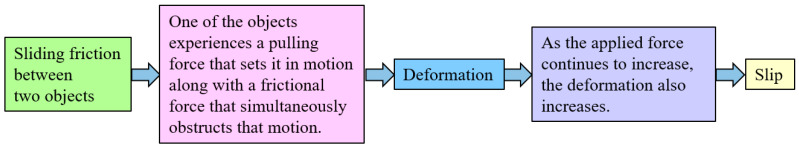
Process of friction vibration generation.

The approximate process of vibration generated by friction is shown in Figure 3. Friction force (FF) is not a constant but fluctuates with the fluctuation of the motion speed, which can excite vibration of moving parts and generate noise. When FF causes the tension and relaxation of moving parts, it can also excite vibration of moving parts and generate noise. When the excitation frequency is consistent with the natural frequency of the moving part, it creates a noise-producing resonance. In addition, FF can excite the coupled vibration of moving parts. Resonance resulting in vibration noise occurs when the vibration frequency produced by the continuous jumping of an object is equal to its natural frequency. Particularly when the different natural frequencies of the moving parts are close, FF can cause modal coupling, thereby exciting strong vibration and generating substantial noise. In recent years, researchers have analyzed the instability of multi-degree-of-freedom systems based on different frictional characteristics to explain the mechanism of rubber bearing vibration and noise. These frictional vibration mechanisms can generally be classified as stick-slip mechanism, sprag-slip mechanism, negative friction–velocity slope mechanism, and modal coupling mechanism.

### 2.1. Stick-Slip Mechanism

**Figure 4:**
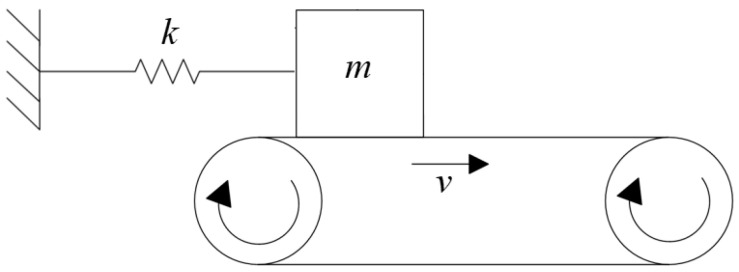
The single-degree-of-freedom model for the stick-slip mechanism.

Stick-slip occurs between two elastic surfaces that are sliding relative to each other. The motion is usually not continuous but intermittent, with a sawtooth waveform for the variation of the FF. It is generally believed that the stick-slip mechanism is caused by the static FC being greater than the dynamic FC. Sinclair et al. [8] proposed the stick-slip mechanism in 1955 and verified it using a single-degree-of-freedom dynamic model. As shown in Figure 4, a mass block m is placed on a conveyor belt with a constant speed. Because the dynamic FC is always smaller than the static FC in the friction pair, in the initial stage, before the FF between the friction pair has reached the static FC, the mass block “sticks” to the conveyor belt; as the displacement of the mass block increases, when the spring’s restoring force is greater than the static FF, the mass block gradually disengages from the conveyor belt, and “slip” occurs, causing the mass block to slide back due to the reverse acceleration; when the restoring force of the spring is balanced with the dynamic FF, the relative sliding between the friction pair stops, at which time the FF between the friction pair takes on the form of static FF, and the above “stick” and “slip” process is repeated. Sticking and slipping alternate, becoming an intermittent and unstable motion that causes self-excited vibration. Kinkaid et al. [9] proposed that there exists a critical relative sliding velocity $v_{c}$ when investigating squeal noise in automotive disc brakes. Stick-slip motion occurs only when the actual relative sliding velocity v is less than $v_{c}$. When v is greater than $v_{c}$, the stick-slip motion disappears.

Bhushan [10] investigated the mechanism of friction noise in water-lubricated rubber bearings (WLRBs) and demonstrated through experiments that the direct cause of noise in the WLRBs is the stick-slip phenomenon that occurs when they cannot form lubricating water film under low-speed heavy loads. According to the frequency range, the noise generated by WLRBs is divided into high-frequency “squeal” and low-frequency “chatter”. Squeal noise is caused directly by the stick-slip motion of the rubber surface, and the corresponding vibration signal does not appear on the surface of its frictional counterpart components. Therefore, the generation mechanism and frequency components of squeal noise are closely related to the rubber lining, and its specific value is affected by the FC between the friction surfaces, the elastic modulus of the rubber material, and the geometrical parameters of the rubber lining. Chatter noise is due to the support structure vibration caused by the stick-slip motion and often occurs at higher loads. It is a low-frequency, high-intensity noise with a frequency equal to the resonant frequency of the rubber-strip support structure and the bearing base. Therefore, the actual frequency of chatter noise is not only affected by the material properties and structure of related components but also by other factors such as specific installation errors and working conditions.

Yao et al. [11] proposed that under heavy load or high water temperature conditions, the interaction between the edge of the water trough and the rotating axis in the rubber bearing is equivalent to the water-wiping action between the windshield wiper and the glass on a car, resulting in friction vibration and emitting a screeching noise. Qin et al. [12,13] conducted tests on the friction pair composed of ZQSn10-2 bronze and rubber using a water-lubricated bearing (WLB) test platform SBB-100. By focusing on the vibration motion image of the rubber layer using a high-speed camera and machine vision technology to extract the friction vibration of the test bearing, the experimental results showed that the bearing vibration is caused mainly by the stick-slip motion. It was found that the mechanism of friction noise in WLRBs may vary under different working conditions, and the weight of each influencing factor may also differ. From strong to weak, the influencing factors were identified as lubrication conditions, contact pressure, hardness, and rubber layer thickness.

Meziane [14] proposed an experimental and numerical analysis on a friction system composed of two contact beams, which induced different types of periodic steady-state vibrations. The results showed that instability caused by friction occurs during self-excited vibration, stick-slip motion, and separation motion on the contact surface. Viswanathan et al. [15] studied the dynamics of the interface of model polymers using coupled force measurement and high-speed in situ imaging technologies to explore the relationships between stick-slip motion and other slow-wave phenomena. They described and explained two new waves (sliding pulse and separating pulse) that are distinct from Schallamach waves [16,17].

Stick-slip motion usually occurs under working conditions that are less than ideal, such as low speed and poor lubrication. Typically, every friction system has a critical relative sliding velocity, and stick-slip motion disappears when the actual relative sliding velocity exceeds the critical value. However, experiments have shown that for high-frequency noise, the location of noise generation does not coincide with the location of stick-slip motion. In practice, high-frequency friction noise usually occurs when the relative sliding velocity is high, which contradicts the concept of the critical velocity of stick-slip motion. Therefore, the mechanism of stick-slip motion cannot reasonably explain the phenomenon of high-frequency friction noise.

### 2.2. Sprag-Slip Mechanism

The theory of sprag-slip was proposed by Spurr in 1961 [18]. Its basic concept is that the self-locking effect between the contact friction surfaces leads to the structural instability of the friction system, which in turn causes friction noise. For a single noise, the noise frequency often varies with changes in the FC, and it is greatly related to the velocity of FC change.

**Figure 5:**
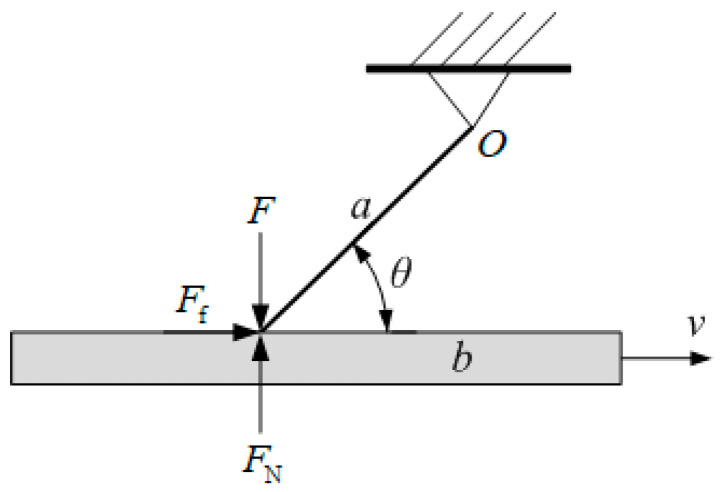
Schematic diagram of sprag-slip mechanism.

The simplified model of the sprag-slip mechanism is shown in Figure 5. Assume that a is an elastic rod with length l, hinged at point O for rotation, and b is a sliding plane with a velocity v relative to the rod in the direction indicated in Figure 5. The angle between the elastic rod a and the sliding plane b is θ, and the rod a is acted upon by F at the contact end. $F_{N}$ represents the normal reaction force, and $F_{f}$ is the FF. An analysis of forces acting on the elastic rod a follows:

(1) $$Fl\cos\theta +F_{f}l\sin\theta -F_{N}l\cos\theta =0.$$

In Equation (1), $F_{f}=\mu F_{N}$. Simplify Equation (1) to obtain the following:

(2) $$F_{f}=\frac{\mu F}{1-\mu \tan\theta }.$$

As can be seen from Equation (2), when μ=cotθ, the FF becomes infinitely large. As cotθ approaches the FC μ, the FF $F_{f}$ will become very large, causing the sliding surface b to be locked.

Sprag-slip is a type of low-frequency self-excited vibration caused by friction. When the contact state is in a certain specific situation, the FF at the contact interface increases rapidly, causing the relative motion between the friction pair to stop and resulting in a self-locking phenomenon. The theoretical foundation of the sprag-slip mechanism is weak, and many friction noise phenomena cannot be reasonably explained by this theory. An example is friction noise, which often occurs in places where the FC is relatively large. In such a case, there is often vibration not only along the friction direction but also perpendicular to the friction direction (tangential direction). Therefore, this theory is relatively less accepted.

### 2.3. The Negative Friction–Velocity Slope Mechanism

**Figure 6:**
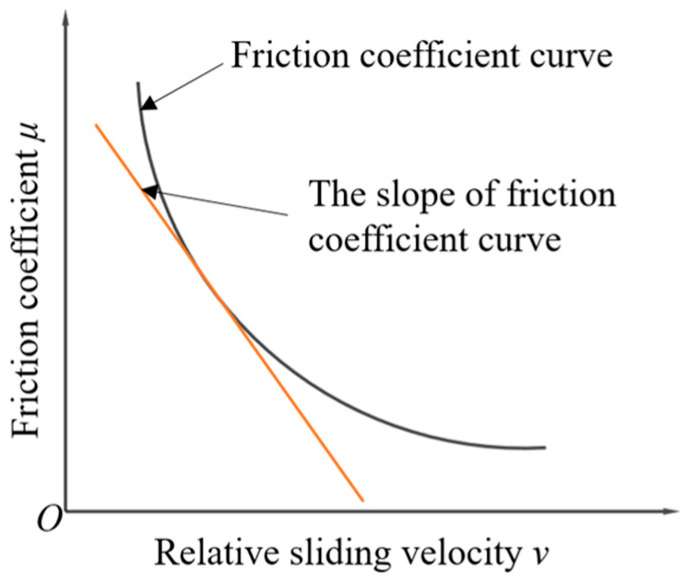
Relationship between FC and relative sliding velocity.

Under insufficient lubrication, such as mixed lubrication, boundary lubrication, and even dry friction states, with the increase in relative sliding velocity between the friction pair, the FF between the two sliding surfaces decreases, and vibration often occurs during this sliding process, resulting in noise. The relationship between FC and relative sliding velocity is shown in Figure 6.

**Figure 7:**
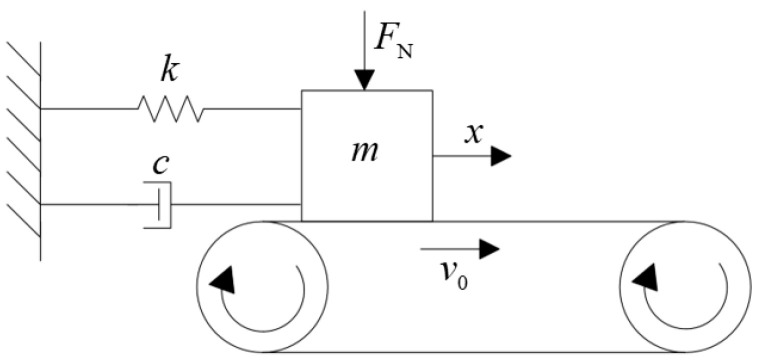
Negative friction-velocity slope mechanism single-degree-of-freedom model.

For a single-degree-of-freedom friction vibration system under the action of negative slope FC, as shown in Figure 7, the control equation is given:

(3) $$m\ddot{x}+c\dot{x}+kx=\mu (v_{0}-\dot{x})F_{N}.$$

In Equation (3), m, c, and k are mass, damping, and stiffness, respectively; $F_{N}$ is the normal force; μ is the FC, and the motion equation is a function of the relative sliding velocity $\left(v_{0}-\dot{x}\right)$.

Perform Taylor expansion on the expression of the FF:

(4) $$\mu (v_{0}-\dot{x})mg=\mu (v)mg-\frac{\partial (\mu (v_{0}-\dot{x}))}{\partial v}\dot{x}mg+\frac{\partial^{2}(\mu (v_{0}-\dot{x}))}{\partial^{2}v}\ddot{x}mg-\cdots .$$

If it is a small-amplitude vibration, higher-order terms can be neglected, and by substituting into the equation, we obtain the following:

(5) $$m\dot{x}+(c+\frac{\partial (\mu (v_{0}-\dot{x}))}{\partial v}mg)\ddot{x}+kx=\mu (v)mg.$$

Let $c_{1}=c+\frac{\partial (\mu (v_{0}-\dot{x}))}{\partial v}mg$, and neglecting the constants unrelated to vibration, we obtain the following:

(6) $$m\ddot{x}+c_{1}\dot{x}+kx=0.$$

Let $x=Ae^{\lambda t}$ and substitute it into Equation (6) to obtain the following:

(7) $$m\lambda^{2}+c_{1}\lambda +k=0.$$

Solving the equation, we obtain the following:

(8) $$\lambda =\frac{-c_{1}\pm \sqrt{c_{1}{}^{2}-4mk}}{2m}.$$

When the slope of the μ−v curve satisfies $c_{1}<0$, the system will exhibit negative damping. Negative damping can cause instability in the friction system, thereby causing intense self-excited vibration of the relative moving components and generating noise.

Krauter [19] conducted experimental simulations of the dynamic interaction between the transmission shaft and the flexible bearing plate lubricated with water. The calculated model predicted the screeching trend of the test device, and the predicted screeching trend was validated by experiments. Simpson [20] established a two-degree-of-freedom nonlinear hydrodynamic bearing dynamics model and used Krauter’s experimental results to numerically simulate the nonlinear response of the FF of the coupled system over time. They believed that the nonlinear variation in the FC with velocity and time led to instability in the system. Peng et al. [21,22] believed that the occurrence of WLRBs noise depends mainly on the direct contact area between the bearing and the shaft neck during the working process as well as the negative slope of the friction-relative sliding velocity curve, which is consistent with Krauter’s conclusion. Chen et al. [23], under reciprocating sliding, found that the degree of friction noise depends on displacement and frequency, and this characteristic could be used to verify the theory of FC decrease causing screeching noise. However, their test showed that the decrease in FC does not always lead to vibration and screeching, and screeching due to friction was also found in many parts where the FC increased.

The theory of negative friction–velocity slope mechanism has been widely accepted in the academic community. However, this theory cannot explain many friction noise phenomena, such as the friction noise sometimes appearing in regions where the slope of the friction–relative sliding velocity is non-negative. In addition, this theory is based on a simple external characteristic model and seriously neglects the stochasticity and temporal variation of friction characteristics. Therefore, it can neither fully explain the complex structure and friction characteristics of brake squeal phenomena nor meet the requirements of design and prediction. Consequently, limitations persist when using this theory to explain the generation of friction noise.

### 2.4. Modal Coupling Mechanism

Modal coupling [24,25] is one of the most important mechanisms for explaining the instability of a friction system. Among the various mechanisms of friction noise, the modal coupling theory can explain many friction noise phenomena. Moreover, based on the modal coupling theory, analysis of many friction noise phenomena can be conducted using finite-element software. Modal coupling refers to introducing FF into a multi-degree-of-freedom system, causing an asymmetric term in the stiffness matrix, causing system instability.

**Figure 8:**
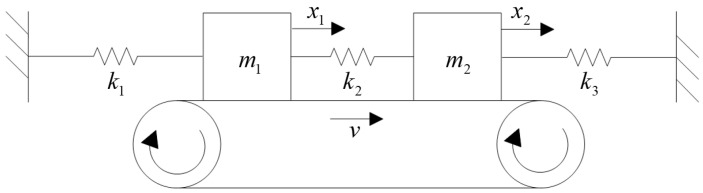
The two-degree-of-freedom model for modal coupling mechanism.

As shown in Figure 8, based on the principles of mechanics, it is easy to establish the dynamic equations of the system using a simple two-degree-of-freedom model:

(9) $$\left\{\begin{array}{c} m_{1}{\ddot{x}}_{1}+k_{11}x_{1}+k_{12}x_{2}=0\\ m_{2}{\ddot{x}}_{2}+k_{21}x_{1}+k_{22}x_{2}=0 \end{array}\right..$$

Here, k is the stiffness coefficient of the system, and when the following conditions are met, the system is stable and will not generate self-excited vibration:

(10) $$\left\{\begin{array}{c} k_{11}>0,\;k_{22}>0\;\;\;\mathrm{Positive}\;\mathrm{stiffness}\\ k_{12}=k_{21}\;\;\;\mathrm{Symmetry}\\ \;k_{11}k_{22}-k_{21}k_{12}>0\;\;\;\mathrm{Positive}\;\mathrm{definiteness} \end{array}\right..$$

Now, assuming that the above system is subjected to excitation forces $-F_{1}$ and $-F_{2}$ and that the excitation forces are, respectively, controlled by the vibration displacements $x_{1}$ and $x_{2}$, we obtain the following:

(11) $$\left\{\begin{array}{c} F_{1}=\lambda_{11}x_{1}+\lambda_{12}x_{2}\\ F_{2}=\lambda_{21}x_{1}+\lambda_{22}x_{2} \end{array}\right..$$

The system neglects its own damping and adopts a displacement-controlled force. Substituting it into Equation (9), we obtain the motion equation of the system:

(12) $$\left\{\begin{array}{c} m_{1}{\ddot{x}}_{1}+k_{11}x_{1}+k_{12}x_{2}+\lambda_{11}x_{1}+\lambda_{12}x_{2}=0\\ m_{2}{\ddot{x}}_{2}+k_{21}x_{1}+k_{22}x_{2}+\lambda_{21}x_{1}+\lambda_{22}x_{2}=0 \end{array}\right..$$

To summarize,

(13) $$\left[\begin{array}{cc} m_{1} & 0\\ 0 & m_{2} \end{array}\right]\left[\begin{array}{c} {\ddot{x}}_{1}\\ {\ddot{x}}_{2} \end{array}\right]+\left[\begin{array}{cc} k_{11}+\lambda_{11} & k_{12}+\lambda_{12}\\ k_{21}+\lambda_{21} & k_{22}+\lambda_{22} \end{array}\right]\left[\begin{array}{c} x_{1}\\ x_{2} \end{array}\right]=\left[\begin{array}{c} 0\\ 0 \end{array}\right].$$

Assuming $K_{\mathrm{ij}}=k_{\mathrm{ij}}+\lambda_{\mathrm{ij}}\left(i,j=1,2\right)$, we obtain the following:

(14) $$\left[\begin{array}{cc} m_{1} & 0\\ 0 & m_{2} \end{array}\right]\left[\begin{array}{c} {\ddot{x}}_{1}\\ {\ddot{x}}_{2} \end{array}\right]+\left[\begin{array}{cc} K_{11} & K_{12}\\ K_{21} & K_{22} \end{array}\right]\left[\begin{array}{c} x_{1}\\ x_{2} \end{array}\right]=\left[\begin{array}{c} 0\\ 0 \end{array}\right].$$

To determine the stability of the system, suppose the general solution of the equation is $x_{1}(t)=A_{1}e^{pt}$ and $x_{2}(t)=A_{2}e^{pt}$, where p=λ+iω represents the complex eigenvalue of the equation. Substituting these equations, we obtain the following:

(15) $$\left[\begin{array}{cc} m_{1}p^{2}+K_{11} & K_{12}\\ K_{21} & m_{2}p^{2}+K_{22} \end{array}\right]\left[\begin{array}{c} A_{1}\\ A_{2} \end{array}\right]=\left[\begin{array}{c} 0\\ 0 \end{array}\right].$$

The condition for the above system of equations to have a non-zero solution is given:

(16) $$\left|\begin{array}{cc} m_{1}p^{2}+K_{11} & K_{12}\\ K_{21} & m_{2}p^{2}+K_{22} \end{array}\right|=0.$$

Expanding further, we obtain the following:

(17) $$m_{1}m_{2}p^{4}+(K_{11}m_{2}+K_{22}m_{1})p^{2}+K_{11}K_{22}-K_{12}K_{21}=0.$$

Suppose $K_{11}>0$, $K_{22}>0$, and $K_{11}/m_{1}=n_{1}{}^{2}>0$, $K_{22}/m_{2}=n_{2}{}^{2}>0$; then, we obtain the following:

(18) $$p^{4}+(n_{1}{}^{2}+n_{2}{}^{2})p^{2}+\left(K_{11}K_{22}-K_{12}K_{21}\right)/\left(m_{1}m_{2}\right)=0.$$

This equation is called the characteristic equation. Solving for it using Equation (18), we obtain the following:

(19) $${\left(p^{2}\right)}_{1,2}=\frac{1}{2}\left[-(n_{1}{}^{2}+n_{2}{}^{2})\pm \sqrt{{\left(n_{1}{}^{2}-n_{2}{}^{2}\right)}^{2}+4\frac{K_{12}K_{21}}{m_{1}m_{2}}}\right].$$

Equation (19) has four roots, which determine the stability and degree of stability of the system.

Let $\Delta ={\left(n_{1}{}^{2}-n_{2}{}^{2}\right)}^{2}+4\frac{K_{12}K_{21}}{m_{1}m_{2}}$.

When Δ>0, the system is a vibratory system with two different mode frequencies.

When Δ≈0, the two mode frequencies of the system tend to converge.

When Δ<0, the two roots of the equation are complex conjugates and can be expressed as given:

(20) $${\left(p^{2}\right)}_{1,2}=-h\pm il.$$

Taking the square root of both sides, we obtain the following:

(21) $$p_{1,2,3,4}=-\alpha +i\omega .$$

In Equations (20) and (21), h, l, α, and ω are all positive real numbers. In this case, the general solution of the equation is given:

(22) $$x_{1}(t)=Ae^{\alpha t}\sin(\omega t)+Be^{\alpha t}\cos(\omega t)+Ce^{-\alpha t}\sin(\omega t)+De^{-\alpha t}\cos(\omega t).$$

The coefficients *A*, *B*, *C*, and *D* in the equation above are determined by the initial conditions of the system. The first two terms are self-excited vibration terms. Over time, the self-excited vibration terms will tend toward infinity, indicating that the system is unstable. This is the explanation of the modal coupling theory.

Mihajlovic et al. [26] analyzed the interaction between vibration and lateral vibration caused by mass imbalance in rotor dynamic test rigs. Numerical analysis showed that two different types of vibrations may occur: torsional vibrations caused by friction and torsional vibrations caused by coupling between torsional and lateral dynamics in the system. Their study also explored the coupling vibration of a flexible rotor induced by friction and objectively proved the existence of a coupling effect between friction, bending, and torsional vibrations. Hua et al. [27] derived the dynamic equations for bending-torsional nonlinear coupling vibration of an imbalanced rotor system using Lagrange equations. They analyzed the vibration characteristics of the coupled system under the action of FF through numerical methods. Li et al. [28] suggested that the vibration between the rotor and bearing is coupled, and its vibration form and amplitude are closely related not only to the bearing design parameters but also to the physical parameters and mass distribution of the rotor as well as to the bearing support position. Kuang et al. [29] investigated the coupling mechanism and process of friction vibration in a rubber, bearing, and shaft system through torsional vibration. High-speed cameras were used to record the response of the rubber, supports, and shaft, while the vibration, noise, friction, and images were analyzed jointly, revealing the friction vibration of the WLB system intuitively. Lin et al. [30] proposed a three-degree-of-freedom modal coupling model considering random rough surface disturbance and subsequently studied the stability of the three-degree-of-freedom system through complex modal analysis.

In addition, deformable bodies have several modal shapes, each with its own frequency. When friction occurs on the surface of an object, coupling phenomena can lead to structural instability, and modes with similar frequencies can couple and produce friction noise at that frequency. However, not all modes with similar frequencies will produce this noise, so this is merely a method for predicting friction noise. Moreover, this theory is based on certain assumptions and special conditions, and it does not take into account nonlinear factors such as the characteristics of the friction interface, which affects friction noise. Therefore, the modal coupling theory can only serve as a linear theory method in the frequency domain, and it cannot analyze time–domain signals or contact behavior of the system during friction motion, which means it offers little help in explaining the underlying mechanism of squealing.

## 3. Friction Noise Suppression Method of Submarine Rubber-Based Propeller Bearings

There are many factors that can cause friction noise in the shaft and propeller bearing, such as water temperature, water quality, bearing structure, bearing materials, surface conditions of the shaft and bearing, alignment between the shaft and bearing, and so on. To suppress abnormal noise in submarine rubber-based propeller bearings, many scholars have conducted related research. This article mainly discusses improvements in the structure and materials of rubber-based propeller bearings.

### 3.1. Structural Optimization

Optimizing the bearing structure and improving the contact state of the bearing surface can enhance the surface lubrication characteristics of the bearing, thereby reducing the generation of abnormal friction noise.

Daugherty et al. [31] investigated seven different flat-strip bearings with the same structure as the U.S. Navy propeller bearings and found that the thickness and shape of the rubber lining significantly influence the dynamic FC. Roy et al. [32] studied a single journal bearing and found through experiments that a thinner rubber layer can achieve better friction performance and furthermore and that the dynamic FC of the flat-strip rubber journal bearing is significantly lower than that of the concave journal bearing.

Zhou [33] analyzed the influence of different groove numbers on the friction noise of water-lubricated rubber alloy bearings and found that a reasonable design of the number of grooves is helpful to reduce the friction noise of the system. Cheng et al. [34] explored the influence of speed and load on a semi-slotted structure and a fully slotted structure. The results showed that under the same working conditions, the semi-slotted structure bearing has a relatively complete structure because the bottom is not slotted, making it easier to form hydrodynamic lubrication. The heat exchange efficiency is high, so the generated heat is easily dissipated by the lubricating water.

Zhou et al. [35] studied the pressure, temperature, velocity, and turbulent energy distribution of the internal flow field of four types of bearings: circular, elliptical, three-leaf, and four-leaf. The results showed that the four-leaf bearing had a smaller negative pressure in the cavity, and the excitation force of the ship was the smallest, which reduced the excitation force outside the ship shafting. Gao et al. [36] designed a new type of bearing with a transition circular arc structure. A transition circular arc structure was added to the bearing to study the influence of different length-to-diameter ratios, rotational speeds, and transition arc angles on the bearing carrying capacity. The results showed that under constant length-to-diameter ratio and angular velocity, the bearing carrying capacity first increases then decreases with the increase in the transition circular arc angle.

Since the 1960s, the development of the propeller bearing structure on U.S. nuclear submarines has gone through three stages. The first stage used metal-backed strip bearings under the MIL-B-17901A standard [37,38], and the second stage used non-metal-backed strip bearings with the MIL-B-17901B standard [31,39]. Both of these stages had problems with abnormal noise due to the gap between the backing and the sleeve. In the 21st century, with the development of the “sea wolf” nuclear submarine, the U.S. Navy conducted extensive research on propeller bearings and finally developed the split-type non-metal-backed half-arc bearing, which was included in the MIL-B-17901C standard [40]. The split-type non-metal-backed half-arc bearing has a composite material shell that effectively absorbs vibration noise. The FC of the inner rubber material is very low; the friction performance and noise characteristics at low speeds are especially excellent.

In recent years, scholars have found that the FC is not necessarily smaller when the surface is smoother. When the friction pair surface presents a certain structure, it has stronger wear resistance and lower surface FC instead [41,42,43]. By setting surface textures on the surface of rubber bearing [44], when the bearing surface is under hydrodynamic lubrication, the additional fluid dynamic pressure generated by the surface textures can increase the bearing capacity; when boundary lubrication is present, the fluid stored in the texture can play a “secondary lubrication” role. Shinkarenko et al. [45] studied the effect of surface textures on the fluid dynamic pressure lubrication of rubber materials and found that when the density and depth of textures are optimized, not only can the bearing capacity be significantly improved, but also the surface friction can be effectively reduced. For soft materials such as rubber, however, surface wear is prone to occur. When the surface textures wear out, the surface lubrication performance of the rubber bearing will be reduced, thereby increasing the probability of abnormal friction noise.

When rubber-based propeller bearings are under low-speed and heavy-load conditions, it is difficult for the bearing surface to form hydrodynamic lubrication, and boundary lubrication exists on the friction interface, which can easily generate friction noise. By introducing hydrostatic lubrication in the contact area, the surface lubrication performance of the bearing can be improved. Hydrostatic lubrication does not require relative motion between the journal and the bearing surface but rather only for lubricating fluid to be pumped into the bearing clearance at a certain pressure to separate the journal from the bearing forcing hydrostatic lubrication.

For hydrostatically lubricated bearings, hydrostatic lubrication is achieved mainly by opening a cavity structure on the bearing surface. Narendra et al. [46] studied the theoretical influence of different cavity shapes on the performance of dynamic and hydrostatic radial sliding bearings. Lin et al. [47] not only studied the cavity shape but also further investigated the influence of cavity depth on the bearing performance. They tested shallow cavities, deep cavities, and step cavities, finding that the step cavity has better performance. Liu et al. [48] set up rectangular cavity discharge outlets at the bow and stern ends of the bearing liner and injected high-pressure water to improve the starting lubrication environment. Wu et al. [49] opened discharge ports on the bearing bushes and connected them with external accumulators through throttling valves. When dynamic pressure lubrication cannot be established at low-speed conditions, high-pressure water can be introduced into the shaft system from the accumulator through the throttling valve to provide forced hydrostatic lubrication. Liu et al. [50] set up circular, elliptical, square, I-shaped, and triangular hydrostatic water cavities near the propeller at the stern end of the bearings and used bearing without water cavities as controls. They used ANSYS/Workbench to perform static analysis on the established model. The results showed that the strain and stress of the bearings with circular cavities on the surface were better than those of bearings with other geometric cavities, and the mechanical performance was best when the circular cavity diameter was between 3–4 mm.

Due to the gravity of the propeller, the local specific pressure of the propeller bearing is relatively high. In order to reduce the local specific pressure and suppress the generation of friction noise, Li et al. [51] designed a magnetic composite rubber-based propeller bearing with a Halbach permanent magnet array. Through stress and strain analysis, the magnetic composite rubber-based propeller bearing showed significantly lower contact stress and surface deformation than ordinary rubber-based propeller bearings under uniform load and tail load conditions.

### 3.2. Material Modification

For some time, a large number of scholars have been committed to developing rubber-based propeller bearing materials with excellent friction and wear performance and low noise. Currently, the main methods for suppressing the frictions and vibration from rubber-based materials include changing the material hardness, surface treatment, and filling reinforcement, imparting self-lubricating properties, and improving hydrophilicity.

The hardness of the rubber strip can have an impact on the friction noise of propeller bearing. Bhushan et al. [10] investigated the influence of rubber-strip material parameters on friction noise and found that rubber with higher hardness is less likely to cause the “stick-slip” effect, and the possibility of noise generation is relatively low. He et al. [52] designed a new type of low-noise WLB material for ships using nanoparticles to modify nitrile butadiene rubber–plastic (NBR–plastic) composite materials and tested the friction properties of the materials with varying hardness in dry and water-lubricated conditions. They found that the material with Shore A hardness of 85 had better friction properties than the material with Shore A hardness of 76 in both conditions. Moreover, the 1:1 prototype of a plate-type WLB manufactured using the material with Shore A hardness of 85 showed no abnormal noise or damage under various test conditions.

Surface treatment is a method of depositing materials with excellent friction performance on the surface of rubber or altering the surface morphology of rubber through chemical means. This can help avert direct contact between the rubber and the friction pair to a certain extent, effectively improving the friction and wear performance of the rubber. Common coating materials include polytetrafluoroethylene (PTFE), diamond-like carbon (DLC), and silicon nitride. Jiang et al. [53] used electron beams to disperse PTFE and polyurethane and their mixtures to make targets and deposited single-layer, double-layer, and composite coatings on the surface of nitrile butadiene rubber (NBR). The FC decreased by more than 50%. Liu et al. [54] deposited Si–DLC coating on the NBR surface and found that Si–DLC coating/NBR exhibits excellent friction and wear performance under low load. Some scholars have also used methods such as XeF2 fluorination modification [55] and sulfonation modification with different concentrations of sulfuric acid [56] to treat the surface of the NBR, all of which can reduce the FC of the NBR.

Filling reinforcement is a method of improving the mechanical properties of composite materials by adding fillers to achieve the purpose of improving rubber friction and wear performance. Commonly used reinforcing fillers include zinc oxide [57], carbon black [58], silica [59], nanoparticles, resins, and short fibers. He et al. [60] first established a three-layer frictional molecular model with an NBR matrix reinforced with nano zinc oxide particles as the core and an iron layer as the counterpart. They concluded that after the incorporation of nano zinc oxide particles, the NBR chains could be effectively constrained around the surface of the nano zinc oxide particles, helping the entire NBR matrix to exhibit better friction and wear performance. Li et al. [61] used phenolic resin to fill and reinforce NBR and found that the addition of phenolic resin improved the friction and wear performance of the composite material, and the mechanical properties of the composite material increased first and then decreased with the increase in phenolic resin dosage. Huang et al. [62] used aramid short fibers to fill and reinforce NBR and found that the addition of aramid short fibers improved the mechanical properties and friction and wear performance of the composite material, and the comprehensive performance of the composite material was optimal when 20 phr was added.

Introducing solid lubricants with low FC into rubber can give the rubber materials self-lubricating properties. This is because as the composite material wears, the solid lubricant gradually appears and forms a lubricating layer on the worn surface, to a certain extent, avoiding direct contact between the rubber and the friction pair, thereby improving the friction and wear performance of the composite material. Some commonly used solid lubricants with excellent performance include graphite, graphene, PTFE, and molybdenum disulfide. Adding a single self-lubricating material alone may not significantly improve the friction or wear performance of the rubber, so two or more self-lubricating materials are often added at the same time, and some self-lubricating materials also have a synergistic effect when added simultaneously.

Wang et al. [63] invented a long-life nitrile-based WLB composite material resistant to mud and sand environments, which uses a synergistic modification composite of solid lubricant, inorganic nanofiller, and organic polymer to effectively reduce the FC and improve the friction and wear performance of the material. Liu et al. [64] used graphene and carbon nanotubes (CNTs) to fill and reinforce NBR and found that graphene and CNTs can effectively reduce the adhesion force and FC of the composite material under water lubrication conditions, and the two-dimensional layered structure of graphene is more likely to precipitate from the NBR matrix to form lubricating water film, effectively reducing the FC. Xin [65] explored the influence of carbon black and molybdenum disulfide content on the wear rate and FC of NBR through experiments and determined that when carbon black was 70 phr, and molybdenum disulfide was 10 phr, the FC of the bearing was minimal, and the volume wear rate was also the lowest. Yang et al. [66] tested the FC, noise, vibration frequency, and amplitude of carbon black/lubricating material/NBR composite materials under low speed and heavy load and found that the additional amount of carbon black and lubricating material will affect the friction performance and FC of the bearing. Wang et al. [67] used a solution-blending method to prepare NBR composite materials filled with three different dimensional carbon nanofillers: CNTs, nanographite, and multilayer graphene. It was found that when the filling amount of the fillers was the same, the three-dimensional nanographite had the best modification effect on the friction and wear performance of the composite material.

Some self-lubricating materials have excellent properties themselves, but due to their poor dispersibility in the NBR matrix, their excellent properties cannot be fully realized, such as graphene and CNTs with a high specific surface area. Jia et al. [68] used solution blending to prepare NBR, NBR/carbon nanotube, NBR/carboxyl carbon nanotube, and NBR/hydroxyl carbon nanotube composite materials. It was found that due to the high strength and high toughness of CNTs, when a certain amount of CNTs were added, the compactness and hardness of the composite material were greatly improved. CNTs can improve the lubrication performance of rubber, and composite materials filled with evenly dispersed carboxyl CNTs have the best friction and wear performance. Zhang et al. [69] studied the friction vibration, friction, and wear properties of graphene/triple-component solid lubricant (graphite/molybdenum disulfide/PTFE)/NBR composite materials. They found that after ionic liquid modification, the dispersion of graphene in the NBR matrix was significantly improved, and it can significantly improve the friction and wear performance while reducing the friction noise of the composite material. Cheng et al. [70] further studied the influence of the mass ratio of graphene to ionic liquid and the content of graphene on the properties of NBR materials. By comparing the FC and the total vibration acceleration level within the 1–5 kHz frequency range of various composite materials, they found that a mass ratio of graphene to ionic liquid of 1:1 was better, and the content of graphene was optimal at 3 phr.

The degree of difficulty for the formation of the lubricating water film between the stern shaft and the bearing is a key factor affecting the FC between them during start/stop and low-speed conditions. Improving the hydrophilicity of the surface of the rubber-based composite material makes it easier for the bearing and the friction pair to form lubricating water film, which can reduce the FC and friction noise of the rubber-based propeller bearing under start/stop and low-speed conditions. He et al. [71] proposed that the hydrophilicity of rubber materials is beneficial to the formation of lubricating water film on the rubber surface, giving it good water-lubricating characteristics. Propeller bearings made of rubber materials with strong hydrophilicity have smaller friction noise. Jiao et al. [72] studied the sliding friction process of rubber under various wettability lubrication conditions and found that the higher the proportion of hydrophilic organic substances in the lubricating fluid, the better the spreading and wettability of the liquid at the interface and the smaller the steady-state sliding FC. Moreover, there is a certain linear correlation between the solid–liquid contact angle and the steady-state sliding FC; the smaller the solid–liquid contact angle, the smaller the steady-state sliding FC. Cheng et al. [73] reviewed the research progress in the surface hydrophilic modification of polymer materials, mainly introducing the three methods of plasma modification, surface coating modification, and surface grafting modification and including a discussion of the prospects of hydrophilic modification of material fillers.

## 4. Conclusions

Rubber-based propeller bearings are widely used in the submarine field due to their many advantages. However, the drawback is that abnormal friction noise can occur during start-up, shut-down, low speed, heavy load, and poor lubrication. In order to suppress the friction noise of submarine rubber-based propeller bearings, many scholars have conducted research on the friction noise mechanism of rubber-based propeller bearings and have successfully suppressed the friction noise by optimizing the bearing structure and improving the bearing material.

The currently matured noise mechanisms include mainly the stick-slip mechanism, the sprag-slip mechanism, the negative friction–velocity slope mechanism, and the modal coupling mechanism. Each mechanism has a complete theoretical system, and the various theories are closely related, but they also have significant differences due to the assorted research objects. In the research field of friction noise from submarine rubber-based propeller bearings, directly adopting a certain mechanism cannot comprehensively explain how friction noise is generated. Therefore, in such research, it is necessary to fully consider all aspects of the actual situation of the friction system to explain the generation mechanism of different friction noises. Then, according to the specific generation mechanism of different friction noises, researchers can optimize the bearing structure and improve the bearing materials to achieve sufficient friction noise suppression.

To further eliminate the friction noise of submarine rubber-based propeller bearings, it is suggested to conduct research in the following five areas:

1. Connect the research on the friction noise mechanism of propeller bearings with the molecular structure of materials to explore the relationship between composite material properties and friction noise at the molecular level;
2. Study the relationships among contact interface micro-asperities, impurities in lubricating water, and friction noise;
3. Study the mechanism of abnormal friction noise of propeller bearings under fluid–solid–heat multi-field coupling and multi-boundary conditions;
4. Research real-time monitoring technology for propeller bearing load distribution, wear status, and friction noise as well as design composite intelligent bearings and develop real-time monitoring and warning diagnostics for the state of propeller bearing;
5. Carry out research on the structural design and control technology of permanent magnet–electromagnetic bearings.

Eliminating friction noise of submarine propeller bearings is of great significance for improving the stealth performance of submarines. With continuous deepening of research, mankind will eventually develop virtually silent submarine propeller bearing.

## Data Availability

The data presented in this study are available on request from the corresponding author.
